# Update on the Mechanisms of Antibiotic Resistance and the Mobile Resistome in the Emerging Zoonotic Pathogen *Streptococcus suis*

**DOI:** 10.3390/microorganisms9081765

**Published:** 2021-08-18

**Authors:** Manon Dechêne-Tempier, Corinne Marois-Créhan, Virginie Libante, Eric Jouy, Nathalie Leblond-Bourget, Sophie Payot

**Affiliations:** 1 Anses Laboratoire de Ploufragan-Plouzané-Niort, Unité Mycoplasmologie, Bactériologie et Antibiorésistance, F-22440 Ploufragan, France; manon.dechene-tempier@univ-lorraine.fr (M.D.-T.); Corinne.MAROIS@anses.fr (C.M.-C.); eric.jouy@anses.fr (E.J.); 2 Université de Lorraine, INRAE, DynAMic, F-54000 Nancy, France; virginie.libante@univ-lorraine.fr (V.L.); nathalie.leblond@univ-lorraine.fr (N.L.-B.)

**Keywords:** *Streptococcus suis*, antibiotic resistance, mechanisms, resistance genes, horizontal gene transfer, competence, conjugation, mobile genetic elements, ICEs, IMEs, GIs, UCSs, prophages, plasmids, biofilm

## Abstract

*Streptococcus suis* is a zoonotic pathogen causing important economic losses in swine production. The most commonly used antibiotics in swine industry are tetracyclines, beta-lactams, and macrolides. Resistance to these antibiotics has already been observed worldwide (reaching high rates for macrolides and tetracyclines) as well as resistance to aminoglycosides, fluoroquinolones, amphenicols, and glycopeptides. Most of the resistance mechanisms are encoded by antibiotic resistance genes, and a large part are carried by mobile genetic elements (MGEs) that can be transferred through horizontal gene transfer. This review provides an update of the resistance genes, their combination in multidrug isolates, and their localization on MGEs in *S. suis.* It also includes an overview of the contribution of biofilm to antimicrobial resistance in this bacterial species. The identification of resistance genes and study of their localization in *S. suis* as well as the environmental factors that can modulate their dissemination appear essential in order to decipher the role of this bacterium as a reservoir of antibiotic genes for other species.

## 1. Introduction

*Streptococcus suis* is a bacterial pathogen causing important economic losses in swine production. *S. suis* colonizes the upper respiratory tract of piglets, in particular the pharyngeal and palatine tonsils, during or soon after pig birth. These early colonization events may lead to an asymptomatic carriage but are also considered the first step for the development of an invasive disease. Symptoms of *S. suis* pig infection have been largely described in the literature over the past 70–80 years and can include arthritis, meningitis, pneumonia, sepsis, endocarditis, encephalitis, polyserositis, abortion in females, and abscesses in growing animals [1,2]. Other animals can also be infected with this pathogen such as ruminants, cats, dogs, deer, or horses [3,4]. Furthermore, *S. suis* can also infect humans and is one of the most common causes of adult bacterial meningitis in South-East Asia (Thailand, Vietnam, China). In Asia, the incidence rate of this pathogen is the highest with up to 0.8 cases per 100,000 population [5]. Sporadic cases have been reported in Western European countries as well, but the ranges are 10 times lower [5]. Cases of *S. suis* human infections have already been observed worldwide. A late diagnostic or an inappropriate or late antibiotic treatment can be fatal, particularly due to a toxic shock-like syndrome [6,7,8]. In pig, *S. suis* is one of the opportunist pathogens of the porcine respiratory disease complex (PRDC), for which the causes of infection are multifactorial [9,10]. In the PRDC, cases of co-infection with other pathogenic bacteria (e.g., *Mycoplasma hyopneumoniae*, *Actinobacillus pleuropneumoniae*, *Bordetella bronchiseptica*, *Glaesserella parasuis*) and/or viruses (e.g., porcine reproductive and respiratory syndrome virus, swine influenza virus, porcine circovirus, porcine respiratory coronavirus) have been documented and generally result in a more severe clinical disease [2,10]. Four major criteria affect swine health: (1) host factors, (2) management inputs, (3) the existence of a stable microbial ecosystem, and (4) facilities [11], and therefore play a role in the infection by this pathogen. The serotype of the bacterium is also a key determinant. Serotypes are defined according to the composition of the extracellular capsule (CPS). Among the 29 already described serotypes, only a few are responsible for most of the diseases caused by *S. suis* [2]. The distribution of serotypes responsible for clinical cases is different between continents [2]. Serotype 2 is found recurrently in North America, South America, Asia, Australia, and Europe. In North America (Canada), serotypes 1, 1/2, and 2 seem to be the most frequently found in cases of infections by this pathogen, whereas in South America, a slight difference in the proportions of serotypes is observed, with a dominance of serotypes 1/2, 2, and 3. In Europe, serotypes 2, 4, 7, and 9 predominate [2]. The serotype distribution can also change with time in the same territory. A new very virulent serotype, the Chz serotype, responsible for meningitis in pigs, seems to have emerged in recent years, and 20 new cps loci (NCL) have been identified in non-typeable isolates [12,13]. Strains of *S. suis* can also be classified into three groups according to their pathotypes: (i) a “non-clinical” group for strains originating from the upper respiratory tract microbiota that do not trigger symptoms or cause the death of its host, (ii) a “respiratory” or “possibly opportunistic” group of strains generally sampled in lungs from clinically sick animals or from clinically healthy animals in farms that have suffered from outbreaks of *S. suis*, and (iii) a “pathogenic” or “systemic” group of strains obtained from samples of blood nervous and/or articular systems and identified as the causes of infection [14,15].

## 2. Antibiotics Used to Treat *S. suis* Infections in Pig Production

In pig production, antibiotics are used to eradicate weaning diarrhea, intestinal infections, and respiratory diseases (“Guidelines for the prudent use of antimicrobials in veterinary medicine” UE 2015). The most commonly used antibiotics in swine industry are tetracyclines, beta-lactams, and macrolides [16,17]. Penicillin/amoxicillin can also be used in combination with aminoglycosides, macrolides, lincosamides, fluoroquinolones, or tetracyclines. Quinolones are not active against streptococci because of their intrinsic resistance, but fluoroquinolones can be used to treat streptococcal infections [18]. Second-generation (thiamphenicol) and third-generation (florfenicol) amphenicols are approved for treatment of swine respiratory disease in several countries including in Europe (European Medicines Agency: EMEA/CVMP/2316/2005-FINAL January 2005). Even if *S. suis* is not always targeted, it can be exposed to these antibiotics since it is present in the upper respiratory tract of pigs. Amphenicols are largely used in China but less in Europe [17]. The use of antibiotics in veterinary medicine, more or less important depending on the country, has led to an enhanced selection pressure, and resistance of *S. suis* isolates have increased worldwide [18,19].

## 3. Mechanisms of Antimicrobial Resistance (AMR) and Resistance Genes Found in *S. suis*

**Figure 1:**
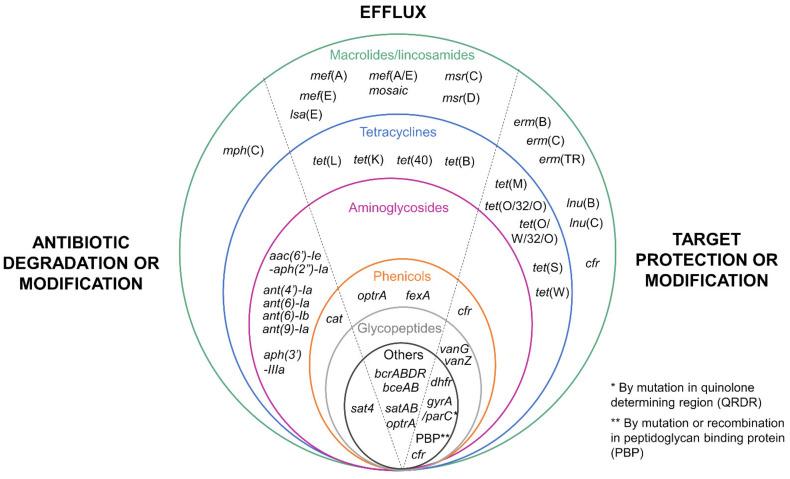
Resistance genes described in *Streptococcus suis*. The different genes conferring antibiotic resistance are classified in two ways: (i) according to their mechanism of resistance (on the left: degradation or modification of the target antibiotic; in the middle: efflux mechanisms; and on the right: target protection or modification, separated by a dotted line) and (ii) according to the family of antibiotics they target (circles of different colors: green for macrolides/lincosamides, blue for tetracyclines, pink for aminoglycosides, orange for amphenicols, gray for glycopeptides, black for other families).

Antibiotics considered in this review have different targets: (i) cell wall inhibitors (such as beta-lactams, glycopeptides, and bacitracin) inhibit the enzymes involved in peptidoglycan synthesis, (ii) protein synthesis inhibitors (such as macrolides, lincosamides, amphenicols, tetracyclines, aminoglycosides, streptothricin, and oxazolidinones) inhibit the elongation of the peptide chain during translation, and (iii) nucleic acid synthesis inhibitors inhibit enzymes (topoisomerase IV, DNA gyrase) involved in DNA replication. According to the mechanism of action of the antibiotic, several mechanisms of resistance can be encountered. For some antibiotic families, up to three different types of resistance mechanisms (antibiotic degradation or modification, efflux and target protection or modification) have been described (Figure 1).

**Figure 2:**
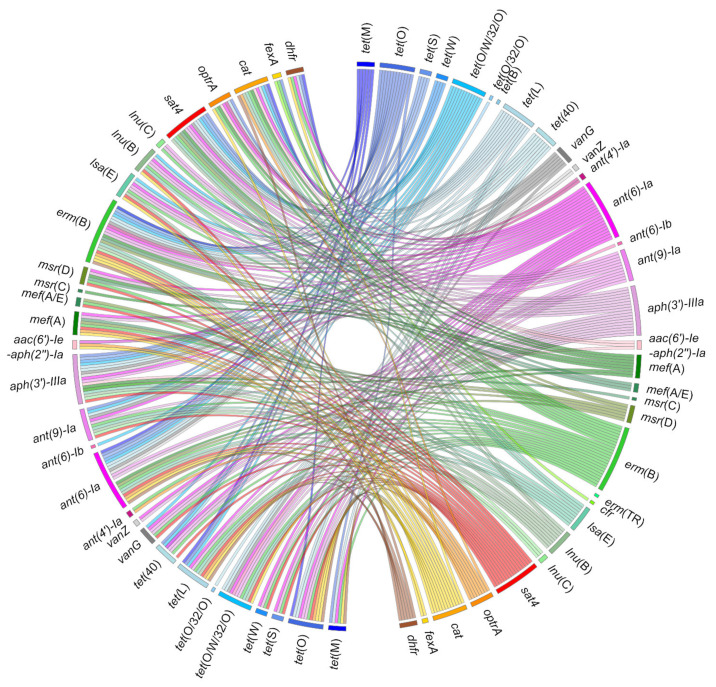
Co-occurrences of resistance genes described in *Streptococcus suis*. Ribbons indicate the co-occurrences between antibiotic resistance genes that have been reported in *S. suis*. The right panel includes all the genes described in *S. suis*. The left panel only includes those that have been described in association with other genes in the same strain of *S. suis*. The color code used is the same as for Figure 1. Detailed data are available as Supplementary Materials (Table S1).

The rate of resistant strains observed in *S. suis* isolates is particularly high against tetracyclines (up to >90%) and macrolides (up to >70%). This is a worldwide phenomenon described for several decades now, and the genetic basis for these resistances has been extensively studied [19]. Many *S. suis* strains resistant to other families of antibiotics have also been described. Multiresistant strains are very frequent, and a huge diversity of resistance gene combinations has been described (Figure 2). The pattern of multiresistance can be impressive—up to 15 resistances observed in strain R61 [20] and 18 in a strain isolated in Spain [21].

### 3.1. Resistance to Tetracyclines

The World Health Organization “WHO” has classified antibiotics according to their use and observed resistance [22]. This classification categorizes tetracyclines as “highly important antimicrobials”. Resistance to tetracyclines in *S. suis* has been widely observed and documented throughout the world with, for example, a percentage of resistant strains ranging from 8% in Sweden (first observation in 1992) to 97% in Brazil (2009–2010 period) [18]. The French surveillance network for antimicrobial resistance in bacteria from diseased animals stated in its annual report of 2019 a resistance rate of 80% to tetracycline for *S. suis* isolated from pigs (RESAPATH 2020).

Two mechanisms of resistance to tetracyclines have been characterized in *S. suis:* (i) efflux pumps that expel the antibiotic outside the cell and are encoded either by *tet*(L), *tet*(B), *tet*(K), or *tet*(40) genes [19,23,24,25,26,27,28,29,30,31] and (ii) ribosome protection proteins, encoded by *tet*(O) (the most frequently described gene in *S. suis*), *tet*(M), *tet*(S), *tet*(W), *tet*(O/W/32/O), or *tet*(O/32/O) genes [18,21,23,24,25,26,30,32,33] (Figure 1).

### 3.2. Resistance to Macrolides and Lincosamides

The WHO has classified macrolides as “critically important antimicrobials of highest priority” [22]. Resistance to macrolides in *S. suis* has also been widely observed, ranging from a rate of 52% in the first observation in Norway in 1986 to 94% in Korea for the 2010 to 2013 period [18]. In France, the annual report of RESAPATH indicated in 2019, 60% of resistance to macrolides and lincosamides (54% for erythromycin, 65% for tylosin, 58% for spiramycin, and 61% for lincomycin) (RESAPATH 2020).

*S. suis* can use two energy-dependent transport systems to counteract the activity of macrolides and lincosamides: (i) ATP-binding cassette (ABC) transporters encoded by *msr*(C) or *msr*(D), and (ii) major facilitator superfamily (MFS) transporters encoded by *mef*(A), *mef*(E), and a mosaic *mef*(A/E) gene [26,30,33,34,35,36]. However, the most frequently resistance mechanisms reported for *S. suis* rely on target site modifications that prevent the binding of macrolides. They are mediated by enzymes that methylate the targets of macrolides, ribosomes, and are encoded by *erm*(B), *erm*(C), and *erm*(TR) genes [21,24,30,34,35]. Another gene encoding a 23s rRNA methyl transferase (*cfr*) has been described to be involved in resistance to selected 16S-membered macrolides [37]. This gene also confers resistance to lincosamides, pleuromutilins, streptogramin A, amphenicols, and the oxazolidinone linezolid [38]. The macrolide phosphotransferases (MPH) enzymes encoded by the *mph*(C) gene phosphorylate macrolides and thereby inactivate them [24] (Figure 1).

Mechanisms of resistance specific to lincosamides have also been reported. The first corresponds to an ABC transporter encoded by the *lsa*(E) gene. This transporter also confers resistance to pleuromutilins and streptogramin A. The second relates to the modification of the target by a nucleotidyl-transferase, encoded by the *lnu*(B) or *lnu*(C) genes [30,35,39,40] (Figure 1).

### 3.3. Resistance to Aminoglycosides

The WHO also classified aminoglycosides as “critically important antimicrobials” [22]. Streptococci naturally exhibit a low-level resistance to aminoglycosides. These antibiotics are currently used in combination with β-lactams in veterinary practice [18]. In France, the annual report of RESAPATH indicated in 2019 a rate of 2% of high-level resistance to aminoglycosides (2% for streptomycin, 4% for kanamycin, 0% for gentamicin). High-level aminoglycoside resistance in *S. suis* is due to several genes encoding aminoglycoside-modifying enzymes that modify the antibiotic compounds, thus leading to their inactivation. The three existing aminoglycoside-modifying enzyme types have been described in *S. suis*. The first one corresponds to aminoglycoside N-acetyltransferases encoded by the *aac* genes with the description of the mosaic *aac(6*″*)-Ie-aph(2*′*)-Ia* gene, mediating high-level resistance to gentamicin and other aminoglycosides except streptomycin. The second type is aminoglycoside O-phosphotransferases encoded by the *aph* genes such as *aph(3*″*)-IIIa*, mediating resistance to several aminoglycosides including kanamycin and neomycin. The third type is aminoglycoside O-nucleotidyl transferases encoded by the *ant* genes such as *ant(4*′*)-Ia* that mediate resistance to several aminoglycosides including tobramycin and amikacin, *ant(6)-Ia* (also called *aadE*), and *ant(6)-Ib* mediating streptomycin resistance or *ant(9)-Ia* mediating spectinomycin resistance [20,24,26,30,33,40,41,42] (Figure 1).

### 3.4. Resistance to Streptothricin

To our knowledge, there is only one streptothricin resistance mechanism described in *S. suis*: a streptothricin N-acetyltransferase encoded by the *sat*4 gene that modifies the antibiotic and inactivates it [33,43] (Figure 1).

### 3.5. Resistance to β-Lactams

Beta-lactams, which notably include penicillin, are a large family of antibiotics, classified as a “highly important antimicrobial” by the WHO [22]. Surprisingly, despite the worldwide use of beta-lactams in pigs for over 50 years, the majority of clinical *S. suis* remain sensitive to these antibiotics. Resistance to penicillin in *S. suis* has been reported in human and pig isolates (ranging from a percentage of 0.3% between 2013 and 2015 in the Netherlands to 26.0% in Spain in a recent report) [8,18,21,44]. Resistance to ampicillin has been reported to be lower than resistance to penicillin or ceftiofur [21,24,45].

In streptococci, beta-lactam resistance can be due to the modification (altered molecular weight and/or decreased affinity for penicillin) of several classes of penicillin-binding proteins (PBP) that are essential for peptidoglycan biosynthesis [18,46]. In 2011, Hu and collaborators made a thorough analysis of the *pbp* mutations that could explain the resistance of the R61 multiresistant strain to cefuroxime (second-generation cephalosporin) and cefotaxime (third-generation cephalosporin). Four PBP proteins have been identified in this strain (PBP2x, PBP2b, PBP1a, and PBP2a) [20]. The highest number of modifications was observed in the PBP2x protein followed by the PBP2b protein. Molecular dynamics simulation later indicated that the modified Ala320, Gln553, and Thr595 residues of PBP2x affect the conformation of the drug-binding pocket and thus induce a reduction of the drug affinity for the PBP2x penicillin-binding protein [47] (Figure 1).

### 3.6. Resistance to Fluoroquinolones

The WHO has classified quinolones as “critically important antimicrobials of highest priority” [22]. Fluoroquinolone resistance is unusual, and less than 2% of resistance has been typically observed in *S. suis* in Europe [36,48]. However, a high rate of resistance to enrofloxacin (46.6%) and danofloxacin (61.2%) has been recently reported for *S. suis* pig isolates in Spain [21].

In streptococci, DNA gyrase (composed of two subunits, GyrA and GyrB) and topoisomerase IV (made up of ParC and ParE) are the primary targets of quinolone action. A single mutation in the quinolone resistance-determining region (QRDR) of *gyrA* or *parC* can reduce susceptibility to fluoroquinolones [49]. Changes reducing drug binding to the enzyme–DNA complex mostly occur at positions S79 and D83 in ParC and S81 and E85 in GyrA [24,29,48,50], with a probable mutation path ParC79→GyrA85→GyrA81 [51]. In the multiresistant strain R61, high-level resistance (>64-fold increase of MIC compared to susceptible strains) to the second-generation fluoroquinolone levofloxacin and to the third-generation fluoroquinolone gatifloxacin is associated with two modifications in GyrA (at positions S81 and E85 of GyrA) and one in ParC (at position S79) [20]. The second mechanism relies on an efflux system belonging to the ATP binding cassette family of transporters. It is encoded by the *sat*(A) and *sat*(B) genes and can extrude the norfloxacin and ciprofloxacin fluoroquinolones [52] (Figure 1).

### 3.7. Resistance to Glycopeptides

The WHO also classified glycopeptides as “critically important antimicrobials of highest priority” [22]. Vancomycin is among the last-resort antimicrobial agents in the treatment of multidrug-resistant Gram-positive bacterial infection. Glycopeptides bind to the end of the growing peptide chain D-Ala-D-Ala, leading to interferences with the activity of the PBP enzymes such as transpeptidases. Vancomycin resistance relies on the modification of the target site. This modification is due to the presence of the *vanG* gene [29] that codes for peptidoglycan precursors with C-terminal modifications. Normally peptidoglycan is terminated by an amino acid sequence D-Ala-D-Ala whereas *vanG* codes for a C-terminal D-Ala-D-Ser amino acid sequence. The replacement of D-Ala by D-Ser induces a reduction of the vancomycin affinity for the peptidoglycan. Resistance to vancomycin linked to the *vanG* locus has been reported in strains of *S. suis* isolated in China [29,42]. A *van*Z-like gene called *van*Z_ss_ that can confer resistance to teicoplanin has also been described in *S. suis* [30,53] (Figure 1).

### 3.8. Resistance to Amphenicols

The WHO classified phenicols (recently renamed as amphenicols) as “highly important antimicrobials” [22]. Amphenicol resistance in *S. suis* is scarce in Europe [21,35,36,54] compared to the rates observed in Asia (up to 41% of resistance) [24,43,55,56]. Amphenicol resistance is mediated by several mechanisms in *S. suis*. The first is the Cfr rRNA methyl-transferase encoded by the *cfr* gene as described above [37], and the second is a chloramphenicol acetyl-transferase mediated by the *cat* gene [57]. *S. suis* antimicrobial resistance genes also encode efflux proteins of the major facilitator superfamily that transport amphenicol-chloramphenicol outside the bacteria, in particular the FexA protein composed of 14 transmembrane helices [24,37,44,58]. *S. suis* can also harbor the *optr*A gene that encodes an ATP-binding cassette (ABC) transporter [44,57,59] (Figure 1).

### 3.9. Resistance to Bacitracin

Bacitracin is a polypeptide produced by *Bacillus licheniformisis* acting on Gram-positive bacteria. The WHO classified cyclic polypeptides as “important antimicrobials” [22]. Bacitracin was previously used as a growth-promoting supplement in animal feed [60]. Several efflux pumps conferring resistance to bacitracin have been described in *S. suis:* (i) the membrane transporter BceAB [60], (ii) the efflux pump SstFEG [60], and (iii) an ABC transporter encoded by the *bcrABDR* genes [61] (Figure 1).

### 3.10. Resistance to Oxazolidinones

Oxazolidinones, in particular linezolid, show high efficiency against most Gram-positive bacteria including pathogens of importance for human health such as vancomycin-resistant enterococci and methicillin-resistant *Staphylococcus aureus*. These antibiotics have been classified as “critically important antimicrobials” by the WHO [22]. Two different genes conferring resistance to these antibiotics have been detected in *S. suis*. The first one is the *cfr* gene (mentioned earlier as it also confers resistance to amphenicols) that encodes a rRNA methyl-transferase [37]. The second one is the *optrA* gene encoding an ABC transporter (also previously mentioned as it confers resistance to several antibiotics including amphenicols and lincosamides) [44,57,59,61]. These genes should be scrutinized with attention due to their potential transfer to pathogens of importance for human health.

## 4. Dissemination of AMR Genes through Horizontal Gene Transfer in *S. suis*

Most of the resistance mechanisms described above are encoded by antibiotic resistance genes and can be transferred to other bacteria through horizontal gene transfer (HGT). By comparison with vertical gene transfer, consisting in genetic information transfer between a cell and its two-daughter cells, HGT enables the acquisition of exogenous genes between distantly related cells [62].

The three most described mechanisms of HGT are transformation, conjugation (through conjugative mobile genetic elements such as plasmids and integrative conjugative elements), and transduction (through phages) [62,63]. More recently described membrane vesicles could also participate to HGT [64].

### 4.1. Horizontal Gene Transfer by Transformation in S. suis

Competence is defined by two natural abilities of the bacteria: (i) the acquisition of naked DNA from the environment and (ii) the integration in their genome [65]. Competence was observed in different bacterial species, but its triggering and regulation have not been deciphered for all [66]. The first evidence of *S. suis* competence induction was reported by Zaccaria and collaborators in 2014 [67]. The *S. suis* transformation-signaling cascade can be divided into several steps. The first step consists of the internalization of an extracellular peptide by a membrane transporter (Opp). The mature peptide derives from a secreted peptide synthesized by the *comS* gene. Once internalized in the cell, the peptide (called a pheromone) interacts specifically with a transcriptional regulator (ComR). This specific interaction is the basis for the definition of different pherotypes. Three have been described thus far in *S. suis* [68]. The interaction of ComR with the pheromone activates the regulator that in turn activates the transcription of the *comX* gene that encodesthe σ^X^ sigma factor. The latter controls the transcription of late competence genes encoding the transformasome competence machinery. The transformasome includes a multi-protein complex involved in the assembly of a type IV-like pilus (encoded by the *comY* operon in *S. suis*) and proteins involved in DNA transport (ComEA, EC, FA, and FC) [69]. After entry into the cells, foreign DNA is taken over by DNA-binding proteins and the homologous recombination system. A fratricidal protein called CrfP regulated by ComR and ComX has been described recently in *S. suis* [70]. This protein is an exported murein hydrolase composed of one N-terminal CHAP domain capable of lysing the target cell and two SH3b domains capable of recognizing and binding the cell wall of *S. suis*. Inactivation of this protein reduced the transformation rate [70]. The biological function of CrfP is likely to lyse and release DNA from related *Streptococcus* strains to provide homologous DNA to competent recipient cells.

Transformation is expensive in terms of energy, and therefore mechanisms of competence shut down exist. In streptococci, shut down occurs through post-transcriptional regulation through a σ^X^ degradation system or by shutting down *comX* expression. None of these mechanisms have yet been characterized in *S. suis* [69].

Competence in streptococci is induced by environmental stresses such as DNA damage, nutrient limitation, or the presence of antibiotics [65,71,72]. Activation of *S. suis* transformation was observed during growth in active porcine and human sera [73]. Transformation is random in the types of genes integrated to the recipient cells. Since DNA integration can be deleterious for the bacteria, long-term maintenance of the transformed alleles will be determined by the ratio of beneficial vs. deleterious genes [65]. DNA acquired by transformation can include mutated chromosomal copies of topoisomerase IV or gyrase genes (conferring fluoroquinolone resistance) or *pbp* genes (conferring resistance to beta-lactams), single or multiple (if grouped on genetic elements) resistance genes, or even whole genetic elements (either mobile or defective ones) carrying AMR genes. These events provide a selective advantage in case of exposure to these antibiotics.

### 4.2. Horizontal Gene Transfer by Conjugation in S. suis

The most frequent mobile genetic elements carrying AMR genes in *S. suis* are integrative and conjugative elements (ICEs) and integrative and mobilizable elements (IMEs) [25,30,33]. Both kinds of elements are chromosomal genetic elements that transfer by conjugation after excision from the chromosome. ICEs can transfer autonomously using a conjugation machinery that they encode. DNA is taken over by a complex called a relaxosome made up of a relaxase and other proteins [74]. The relaxase of this complex is recognized by a coupling protein (CP), which will then interact with the type IV secretion system (T4SS) present in the cell wall that enables the translocation of the DNA–relaxase complex into the recipient cell. IMEs are not autonomous and rely on the conjugation machinery of autonomous conjugative elements (ICEs or plasmids) for their transfer [75]. IMEs are poorly studied and thus overlooked, but likely play a major role in the transfer of resistance genes in *S. suis* [30]. Many studies carried on *S. suis* genomes do not include a proper search of ICEs and IMEs since the available bioinformatic tools are usually not efficient and cannot cope with the presence of multiple related elements in the same strain, internal accretions, elements in tandem, and mosaic patterns (between conjugative elements but also with prophages) observed in this bacterial species.

ICEs and IMEs can be integrated in different sites depending on the recombination enzyme (called integrase) they encode: tRNA genes, ribosomal protein-encoding genes, and other conserved genes, some of them being located inside other mobile genetic elements. ICEs and IMEs are delimitated by attachment sites (*attL* to the left and *attR* to the right) that enable integrase binding and recombination, allowing integration or excision of the genetic element.

**Figure 3:**
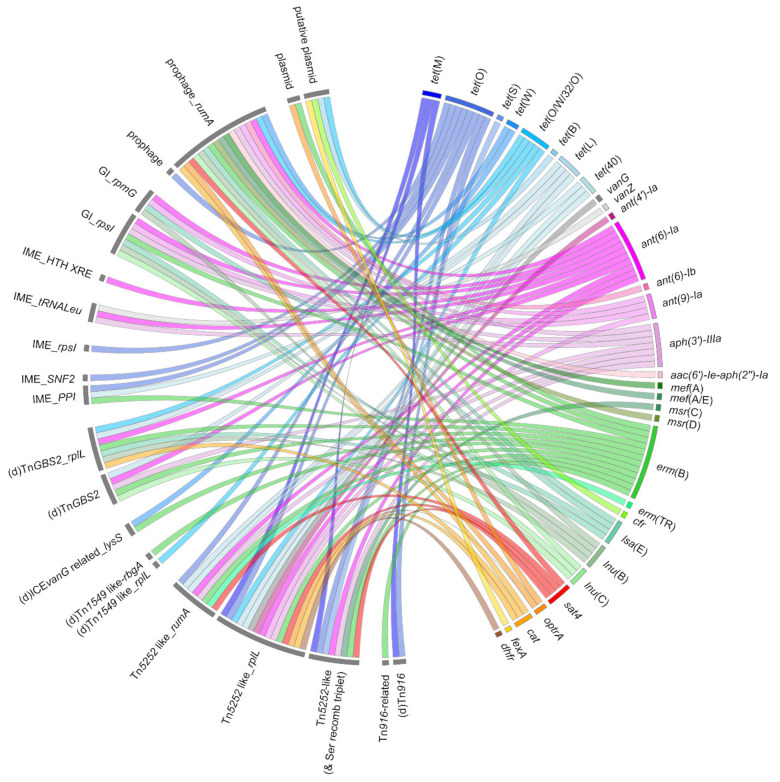
Mobile genetic elements carrying resistance genes described in *Streptococcus suis*. Ribbons indicate the association between resistance genes (**appearing in the right panel**) and mobile genetic elements (**on the left panel**). The color code used to distinguish the different resistance genes is the same as for Figure 1 and Figure 2.

Five ICE families (defined by their conjugation genes) carrying AMR genes have been described in *S. suis*: elements of the Tn*5252*, Tn*1549*, and Tn*916* families; elements related to Tn*GBS2*; and ICEs integrated in the *lysS* gene related to ICE*vanG*. These various ICEs carrying AMR genes are presented in Figure 3 [25,26,29,30,33,42,76,77]. Until now, only ICEs of the Tn*5252* family have been studied experimentally to evaluate their mobility in *S. suis* [12,23,25,30,33,57,77].

ICEs can integrate into a site already harboring a genetic element leading to the formation of a tandem. Two types of tandems have been described thus far: ICE–ICE tandems and ICE–prophage tandems [26,33]. ICEs can also integrate inside other ICEs (internal accretions), e.g., Tn*916* and Tn*5397* integrations inside Tn*5252*-related ICEs [26,33].

ICEs can also host IMEs carrying antibiotic resistance genes. This is the case for IMEs integrated in the SNF2 (encoding a putative helicase) and PPI (peptidylprolyl isomerase) genes of ICEs of the Tn*5252* family that carry *tet*(O) or *tet*(O)-*erm*(B) genes [30].

Several other putative IMEs carrying AMR genes have been identified in *S. suis*: IME_*rpsI* carrying *tet*(O) (tetracycline resistance), IME_HTH-XRE with the *ant(6)-Ia* gene (streptomycin resistance), and IME_*tRNALeu* carrying *aph(3′)-IIIa*-*van*Z-delta *sat4*-*ant(6)* genes conferring resistance to kanamycin, teicoplanin, streptothricin, and streptomycin respectively (Figure 3) [30]. Like ICEs, IMEs can form tandems [30]. Further studies are needed to identify the conjugative elements that enable the mobilization of these IMEs carrying AMR genes.

Genomic islands (GIs) integrated in the *rpsI* gene have been mis-annotated as ICEs, even if they lacked conjugation genes [40]. These elements could be mobilizable by diverting the necessary elements of an ICE. Several resistance genes were located on GIs integrated in *rpsI*: *ant(9)*-*erm*(B), *ant(9)-lnu*(B), *ant(9)*-*lnu*(C),* ant(9)*-*erm*(B)-*lnu*(B), *ant(9)*-*lnu*(C)-*erm*(B), *ant(9)*-*lnu*(B)*-lsa*(E)*,* and *ant(9)*-*aph(3′)-IIIa*-*lnu*(B)*-lsa*(E)*-ant(6)*, conferring resistance to macrolides, lincosamides, and aminoglycosides (Figure 3) [30].

Unconventional circularizable structures (UCSs) are genetic elements that lack recombinase but can excise as a circular form thanks to extensive direct repeats (DRs) flanking the DNA segment [78]. In *S. suis,* UCSs can carry multiple antibiotic resistance genes *tet*(O/W/32/O), *tet*(40), *erm*(B), *ant(6)-Ia*, and *aph(3″)-IIIa* [79]. By targeting a conserved site on ICEs of the Tn*5252* family that transfer at high frequency, these genetic elements may employ a parasitic strategy to spread among the streptococcal population [79].

Until now, there has been no description of conjugative (or even mobilizable) plasmid carrying AMR genes in *S. suis*. Some AMR genes (*erm*(B) and *tet*(O/W/32/O, *tet*(B), *cfr* or *optrA*) could be carried by non-self-transmissible plasmids, but further studies are needed for most of them to confirm these results (Figure 3) [37,79,80,81]. If they are carried by transposons, these genes may move intracellularly or gain intercellular mobility by integrating in conjugative elements.

### 4.3. Horizontal Gene Transfer by Transduction in S. suis

Bacteriophages can be integrated into the bacterial chromosome as prophages. After entry into lytic phase, bacterial DNA can be fortuitously packaged in the viral capsid and be transferred into another cell. This process is called transduction. In 2008, Ma and Lu were the first to demonstrate the existence of a lytic bacteriophage (bacteriophage SMP) capable of infecting *S. suis* [82]. In 2015, a genomic study of 375 *S. suis* genomes highlighted five genes presenting sequence homology with genes of a lytic bacteriophage [14]. In 2017, Huang and collaborators described the *optrA* resistance gene carried by a prophage (Φm46.1) [59]. This prophage belongs to a family originally described in *S. pyogenes* and *S. agalactiae*, thus suggesting the ability of this prophage to infect other streptococcal species. Resistance genes such as *erm*(B), *mef*(A), *aph(3*′*)-III*, *ant(6)-Ia*, *aac (6′)-aph(2″)*, *cat*(pC_194_), *optrA*, *sat4*, *tet*(O/W/32/O), and *tet*(W) were also observed in prophages (Figure 3) [40,42,57]. ICEs and prophages can also be found together in chimeric and tandem forms [26].

## 5. Formation of Biofilm and Antimicrobial Resistance

Bacteria are present in two forms in the environment, in planktonic form (isolated in suspension in a liquid medium) and in sessile form in contact with a surface (forming a biofilm). The development of bacteria in biofilms is a major survival strategy. In a biofilm, bacteria aggregate into a complex structure, embedded in an extracellular matrix also including protozoan and micro-invertebrate communities. This matrix contains an extracellular polymeric substance (EPS) secreted by the bacteria and mainly composed of polysaccharides, extracellular DNA, proteins, and cellulose [83]. The biofilm confers protection to the bacterial communities colonizing these surfaces against antibiotics [84] and biocides [83]. Biofilm development of *S. suis* increases its resistance to penicillin and ampicillin [85].

Within this biofilm and generally in the environment, the bacteria communicate with each other through quorum sensing. Quorum sensing is an intercellular communication system used by many bacteria including *S. suis* [77,86,87,88]. When the concentration of signaling molecules, such as the LuxS autoinducer-2, reaches a threshold level, bacteria can induce the expression of genes that participate to diverse processes, in particular, biofilm formation and antibiotic resistance. Strains with a LuxS quorum sensing system showed an increased susceptibility to fluoroquinolones [87], while addition of exogenous AI-2 signaling molecule increases resistance to tetracycline though a regulatory effect on Tn*916* carrying *tet*(M) [89]. Other molecules, in particular sub-inhibitory concentrations of amoxicillin, lincomycin, and oxytetracycline, were described in the literature to induce biofilm formation [77,90]. In 2020, Wang and collaborators suggest a possible cooperation between *S. suis* and *Actinobacillus pleuropneumoniae* [88]. They found an increase in antibiotic resistance when the two bacteria are co-cultured in biofilms. Different mechanisms are responsible for antibiotic resistance of *S. suis* embedded in biofilm: high expression of EPS, efflux pumps, reduced growth and metabolic adaptation, stress response induction, high probability of gene mutations, and horizontal gene transfer [77].

## 6. Conclusions

*S. suis* is a bacterial pathogen responsible for severe infections in pigs and consequently causes animal suffering and important economic losses in pig production. This bacterial species can also colonize other animal hosts, including wild fauna, and is a zoonotic pathogen for humans. A plethora of antibiotic resistance genes have been described in this bacterium, most of them being carried by mobile genetic elements (in particular ICEs, IMEs, and prophages). It is worrying since *S. suis* can serve as a reservoir of resistance genes to other species sharing the same habitats. It is therefore essential to further characterize the mobile resistome of this pathogen that can compromise animal and human health and participate to the dissemination of AMR genes between animals and humans. This is one of the worldwide challenges to be taken up in the incoming years in order to preserve the activity of critical antimicrobials.

## Supplementary Materials

The following are available online at https://www.mdpi.com/article/10.3390/microorganisms9081765/s1, Table S1: Associations between antimicrobial resistance genes reported in *Streptococcus suis.*
